# Outcomes of both abbreviated hyper‐CVAD induction followed by autologous hematopoietic cell transplantation and conventional chemotherapy for mantle cell lymphoma: a 10‐year single‐centre experience with literature review

**DOI:** 10.1002/cam4.543

**Published:** 2015-10-03

**Authors:** Turki Abdulaziz Alwasaidi, Abdulaziz Hamadah, Sultan Altouri, Jason Tay, Sheryl McDiarmid, Carolyn Faught, David Allan, Lothar Huebsch, Christopher Bredeson, Isabelle Bence‐Bruckler

**Affiliations:** ^1^ Ottawa Hospital Blood and Marrow Transplant ProgramOttawaOntarioCanada; ^2^ College of Medicine at Taibah UniversityAlmadinah AlmunawarhSaudi Arabia; ^3^ Department of Medical OncologyCancer CenterKuwaitKuwait; ^4^ Ottawa Hospital Research InstituteOttawaOntarioCanada

**Keywords:** Autologous hematopoietic cell transplantation, conventional chemotherapy, mantle cell lymphoma, nontransplant eligible

## Abstract

We retrospectively evaluated consecutive patients diagnosed with Mantle cell lymphoma (MCL) between 01 January 2000 and 31 December 2009. Eighty eight patients with MCL were included in the analysis of whom 46 (52%) received abbreviated Hyper‐CVAD (a total of two cycles; with addition of Rituximab since 2005) with an intention of proceeding to autologous hematopoietic cell transplantation (auto‐HCT), with a median age of 58 years. Response rate to induction at auto‐HCT time was 89% and complete response was 61%. Forty four patients received an auto‐HCT with a 5‐year progression‐free survival (PFS) and overall survival (OS) were 31.2% and 62.5%, respectively. There were 42 nontransplant eligible patients with a median age of 72 years, and 5‐year PFS and OS were 0.0% and 39.9%, respectively. The median survival and PFS in the auto‐HCT eligible group were 68 and 33 months, compared to 32 and 12 months in nontransplant eligible group, without a plateauing of the survival curves in either group. Treatment‐related mortality in the auto‐HCT eligible group was 10.9% (*n* = 5); two patients died during R‐Hyper‐CVAD and 3 (6.8%) experienced transplant‐related mortality. An abbreviated R‐Hyper‐CVAD‐based induction strategy followed by consolidative auto‐HCT is feasible and provides moderate potential of long‐term survival. Further research to define risk‐adapted strategies; to optimize disease control, is required.

## Introduction

Mantle cell lymphoma (MCL) has a variable course. A minority of patients may survive untreated for many years, however for most, the disease will follow an aggressive course 1. Patients with MCL are typically older with a male predominance and present with stage IV disease 2. MCL cells are characterized as CD20^+^ CD5^+^ CD23^−^ with t (11,14) (q13; q32) translocation and cyclin D1 overexpression on immunohistochemistry 1, 2. Overexpression of cyclin D1 disrupts cell cycle regulation by increasing retinoblastoma protein phosphorylation, leading to the loss of its inhibitory effect on the G1/S cellular transition phase. The detection of cyclin D1 by immunohistochemistry permits distinction of this disease from other lymphoproliferative disorders that might appear morphologically similar 3.

The role of autologous hematopoietic cell transplantation (auto‐HCT) in MCL has been controversial. Most studies that support the utility of this approach are small phase II single‐institution studies with highly selected patient populations 3. The effect of pretransplant induction regimens on the outcomes of auto‐HCT as the first line treatment is being defined. Recent data reveal that including high dose cytarabine in the pretransplant induction regimen improves outcomes of response rate, disease‐free survival, and even overall survival (OS) 4, 5, 6, 7, 8, 9. We retrospectively evaluated the long‐term outcomes of patients diagnosed and treated at The Ottawa Hospital over a 10‐year period.

## Methods

### Patients

Patients with previously untreated MCL between 01 January 2000 and 31 December 2009 were identified retrospectively using the Ottawa Hospital Blood and Marrow Program database and Pathology laboratory database. Transplant eligible patients received abbreviated Hyper‐CVAD with an intention of proceeding to consolidative high dose chemotherapy and rescue with auto‐HCT. Outcomes were analyzed for the patients who were not eligible for auto‐HCT because of age (>65 years) and/or comorbidities, treated during the same time period. The treatment approach in our institution for patients was based on the physician discretion for transplant eligibility or ineligibility that depend on patient age and comorbidities. Research Ethics Board of The Ottawa Hospital Research Institute approved this study.

### Treatment

Patients who were eligible for auto‐HCT received two cycles of Hyper‐CVAD as first line induction chemotherapy. Parts A (cyclophosphamide 300 mg/m^2^ q12 h—Day 1, 2, 3; vincristine 2 mg—Day 4, 11; doxorubicin 50 mg/m^2^—Day 4; and dexamethasone 40 mg—Day 1, 2, 3, 4 and 11, 12, 13, 14) and B (high‐dose methotrexate 1 g/m^2^—Day 1 and cytarabine 3 g/m^2^ q12 h—Day 2, 3; MA) were considered one cycle. Rituximab (375 mg/m^2^) was included in the induction regimen, with both parts A and B, as of January 2005. Hematopoietic stem cells (HSCs) were mobilized and collected from peripheral blood, using cyclophosphamide (CY) and filgrastim (Granulocyte‐colony stimulating factor) between cycles one and two of Hyper‐CVAD. The collected HSCs were infused following conditioning with BEAM (BCNU, etopside, cytarabine, and melphalan) in most of the patients and alternative conditioning regimens were a combination Melphalan, VP16 (etopside) and total body irradiation (TBI) and CY and TBI.

**Figure 1:**
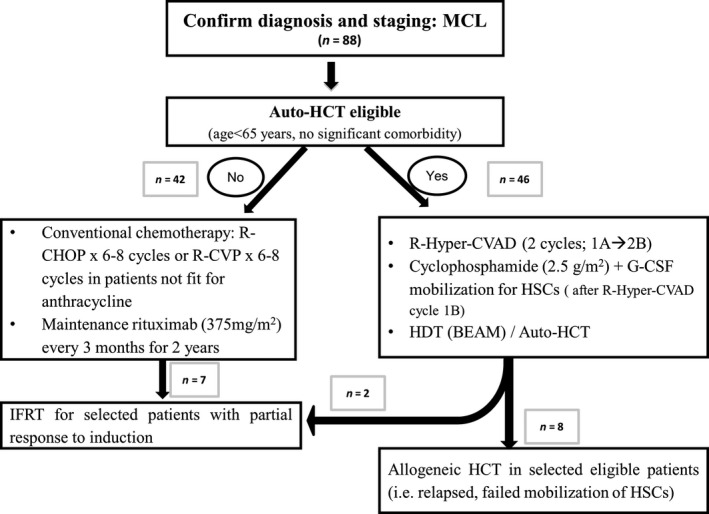
The current Ottawa Hospital approach of managing newly diagnosed MCL patients. Allogeneic HSCT, allogeneic hematopoietic stem cell transplant; Auto‐HCT, autologous hematopoietic stem cell transplant; BEAM regimen, BCNU, etopside, cytarabine, and melphalan; HDT, high dose therapy chemotherapy; HSC, hematopoietic stem cells; IFRT, involved field radiation therapy; MCL, mantle cell lymphoma; R‐Hyper‐CVAD, parts A (rituximab, cyclophosphamide, vincristine, doxorubicin, and dexamethasone) and B (rituximab, high‐dose methotrexate and cytarabine) were considered as one cycles of therapy.

Most patients not eligible for auto‐HCT due to advanced age >65 years and/or comorbidities were induced with six to eight cycles of R‐CHOP (rituximab 375 mg/m^2^—Day 1, cyclophosphamide 750 mg/m^2^—Day 1, adriamycin 50 mg/m^2^—day 1, vincristine 2 mg—Day 1, and prednisone 100 mg—Day 1, 2, 3, 4, 5), or R‐CVP (rituximab, cyclophosphamide, vincristine, and prednisone). Maintenance rituximab (MR) (375 mg/m^2^ every 3 months for 2 years) was added to treatment protocols in 2005 for most patients not eligible for auto‐HCT (Fig. 1).

### Definition of data elements and survival events

Patients were staged using the Ann Arbor staging system. The Mantle Cell Lymphoma International Prognostic Index (MIPI) was calculated for the patients in auto‐HCT group 10, 11. Response was evaluated according to response criteria for malignant lymphoma 1999, proposed by Cheson and colleagues 12, 13. Complete response (CR) was defined by a reduction of the sum of diameter product of nodal and extra‐nodal measurable lesions greater of 75% without (CR) or with (CRu) residual mass more than 15 mm and a normal Bone Marrow (BM) examination. Partial response was defined by a reduction of initial tumor mass greater than 50% but less than 75% and/or persistence of BM involvement. Progression‐free survival (PFS) was measured from diagnosis to disease relapse/progression or death. OS was measured from diagnosis to death from any cause. Treatment‐related mortality (TRM) was defined as death in the absence of disease progression from initiation of induction chemotherapy in both groups. Patients were censored at last follow up.

### Statistical analysis

The Fisher exact test was used to assess the difference between proportions. Five‐year PFS and OS were estimated using the method of Kaplan–Meier. Log‐rank tests were used to evaluate differences in survival. Time‐to‐event analyses were measured from diagnosis date. All statistical tests were 2‐sided with alpha set at 0.05. All statistical analyses were conducted using SAS, Ottawa, Ontario, Canada.

## Results

### Patients

Eighty‐eight patients were included in the analysis of whom 46 (52%) received Hyper‐CVAD with an intention of proceeding to auto‐HCT, with a median age of 58 years (range, 40–65). Demographics and disease characteristics for both groups are provided in Table 1. There were 39 (85%) males in the auto‐HCT eligible cohort, with 37%, 33% and 15% of patients having low, intermediate, and high M‐IPI scores, respectively. Forty‐four patients received peripheral blood derived stem cells mobilized using CY and filgrastim following the first cycle of Hyper‐CVAD. Of these, 41 (93%) received BEAM conditioning, two received a combination of Melphalan, VP16, and TBI and one received CY/TBI.

**Table 1:** Patient demographics and disease characteristics

	Variables	Auto‐HCT eligible group (*n* = 46)	Non‐transplant eligible group (*n* = 42)
Age—median years (range)		58 (range 40–65)	72 (range 63–90)
Gender	Male	39	36
	Female	7	6
	*P* = 0.936		
Stage at diagnosis	I	2	2
	II	1	1
	III	3	8
	IV	40	31
	*P* = 0.935		
B symptoms	A	22	20
	B	24	22
	*P* = 0.293		
MIPI risk score	Low	17	NA
	Intermediate	15	NA
	High	7	NA
	Missing	7	42
Year of diagnosis^a^	2000–2004	19	26
	2005–2010	27	16
	*P* = 0.784		
Radiation therapy with induction		2 (4.7%)	7 (16.7%)
Transplant characteristics	CD34 count (median)	5.47 × 10^6^/kgRange (2.21—26.13)	NA
	Conditioning for auto‐HCT	BEAM 41 (93.2%)Mel/ VP16/TBI 2 (4.5%)CY/TBI 1 (2.3%)	NA
	Allogeneic HCT for relapse of MCL after auto‐HCT	8	NA

HCT, hematopoietic cell transplantation; MIPI, Mantle Cell Lymphoma International Prognostic Index; N/A, not applicable or not available; BEAM, BCNU, etopside, cytarabine, and melphalan; TBI, total body irradiation; CY, cyclophosphamide; MCL, Mantle cell lymphoma.

a Rituximab was included in Hyper‐CVAD induction, with both parts A and B, as of January 2005.

In contrast, 42 patients who did not undergo auto‐HCT had a median age of 72 years (range 63–90). There were no statistically significant difference in gender, year of diagnosis, stage at diagnosis, or B symptoms between transplant eligible and non‐transplant eligible patients (Table 1). Sixty two percent (*n* = 26) received CHOP induction chemotherapy (21 of 26 patients had R‐CHOP; five of them received MR) and 31% (*n* = 13) received CVP induction (nine of 13 patients had R‐CVP; three of them received MR). Furthermore, three patients received only prednisone as palliative therapy due to significant comorbidities, poor performance status, and advanced age. One of these patients had advanced MCL with significant comorbidities and her age was 90 years, the second had advanced MCL with coexistence advanced bladder cancer, and the third was 74 years old with advanced MCL and significant comorbidities, including end stage renal disease, coronary artery disease, and chronic obstructive pulmonary disease (COPD).

### Treatment outcomes

Two of 46 auto‐HCT eligible patients died during Hyper‐CVAD induction. Forty‐four patients who completed two cycles of Hyper‐CVAD underwent successful graft collections using CY and filgrastim following the first cycle of Hyper‐CVAD, with median CD34^+^ counts of 5.47 × 10^6^/kg (range 2.21–26.13 × 10^6^/kg). The mobilization and collection of HSCs were not associated with mortality. Overall response rate (ORR) to induction at auto‐HCT time was 89% (*n* = 41) and CR was 61% (*n* = 28).

TRM in the auto‐HCT eligible group was 10.9% (*n* = 5). Two patients died after part A of the first cycle of Hyper‐CVAD induction; one death was related to perforated abdominal viscus with sepsis and the other was due to status epilepticus with septic shock. The day +100 transplant‐related mortality among the auto‐HCT patients was 6.8% (*n* = 3). Deaths occurred on day +4, +37, and +35 post auto‐HCT and were related to septic shock, septic shock associated with candidemia, and septic shock complicated with myocardial infarction. The median time to neutrophil and platelet engraftment post‐auto‐HCT was 11 days (range 2–25) and 14 days (range 7–36), respectively.

**Figure 2:**
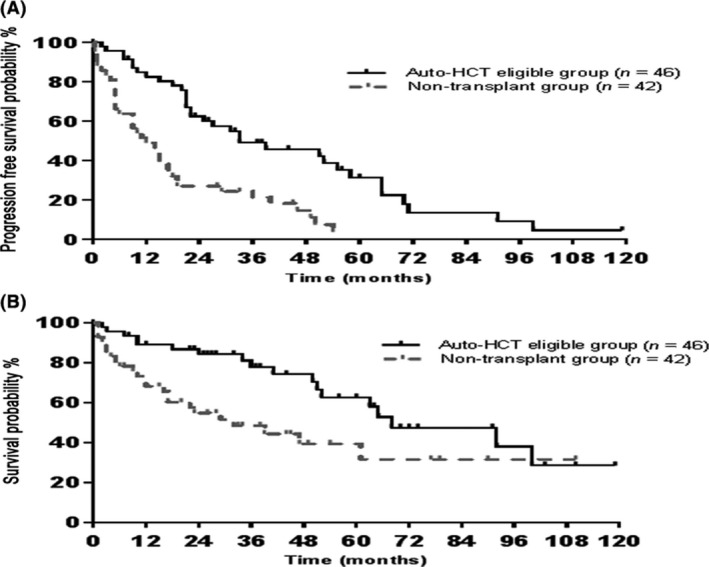
Outcomes by group: (A) 5‐year progression‐free survival (B) 5‐year overall survival.

**Figure 3:**
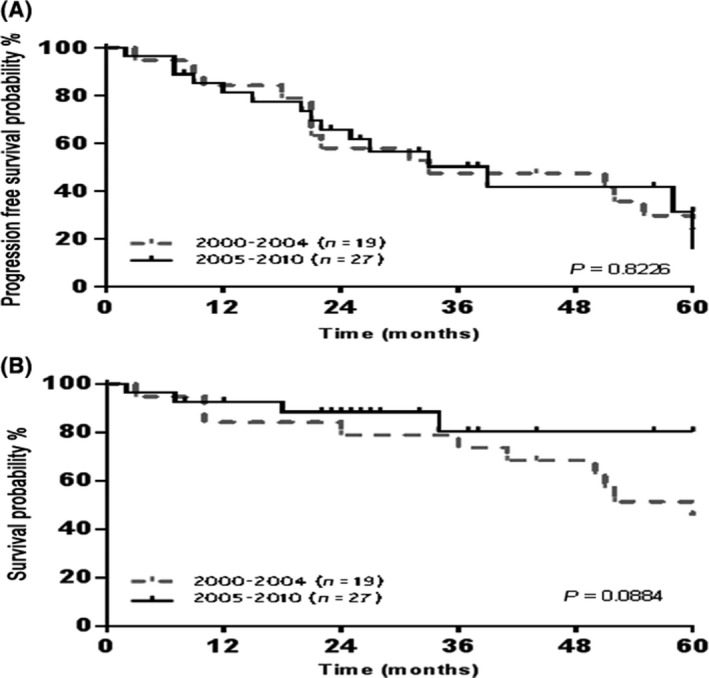
Outcomes related to date of diagnosis among auto‐hematopoietic cell transplantation eligible group: (A) 5‐year progression‐free survival (B) 5‐year overall survival.

**Figure 4:**
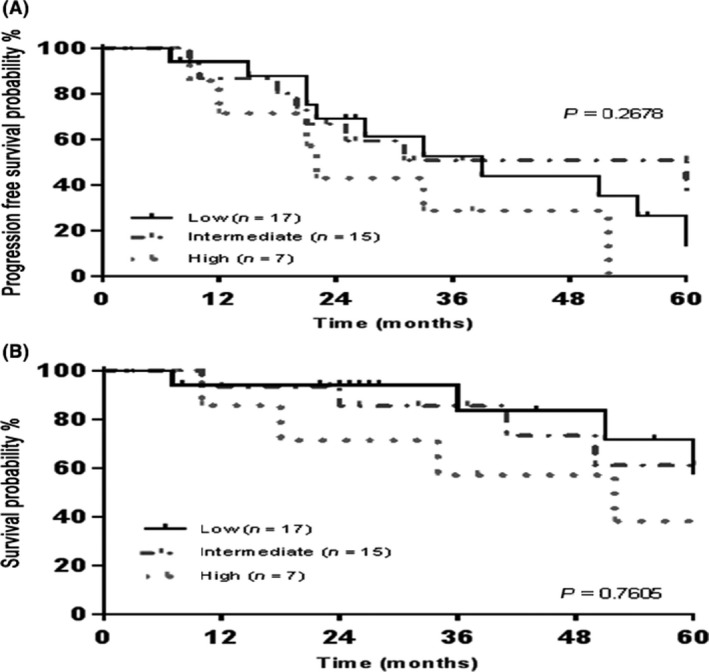
Outcomes related to Mantle Cell Lymphoma International Prognostic Index risk among auto‐hematopoietic cell transplantation eligible group: (A) 5‐year progression‐free survival (B) 5‐year overall survival.

The median follow‐up for all patients was 29 months; 37 months in auto‐HCT group, and 22 months in non‐transplant group. The 5‐year PFS and OS rates in auto‐HCT eligible group were 31.2% and 62.5%, respectively (Fig. 2). There was no difference in OS and PFS among patients receiving an auto‐HCT between 2000–2004 and 2005–2010 (*P* = 0.0884 and 0.8226, respectively) (Fig. 3). Furthermore, there were no OS or PFS differences among patients with low, intermediate or high M‐IPI scores among patients receiving an auto‐HCT (*P* = 0.7605 and 0.2678, respectively) (Fig. 4). Eight patients in the auto‐HCT group underwent subsequent allogeneic HCT for relapsed MCL (age range 41–59 years) after salvage chemotherapy. Three of these patients then relapsed post‐allogeneic HCT and three patients died, due to MCL relapse in two patients and severe bronchiolitis obliterans with organizing pneumonia in one patient. All allogeneic HCTs for relapsed MCL took place after 2005 with a median follow‐up of 9 months (range 4–76).

In contrast, nontransplant eligible patients were older and deemed not eligible to receive intensive regimens because of comorbidities. The TRM among the non‐transplant eligible group was 9.5% (*n* = 4) and all occurred in patients receiving R‐CHOP. Two patients aged 70 years died as a result of neutropenic sepsis following the first and second cycles of R‐CHOP, respectively. The third patient was 73 years old and experienced neutropenic sepsis following second cycle of R‐CHOP complicated by gastrointestinal bleeding. The fourth patient, aged 77, developed neutropenic sepsis complicated by myocardial infarction and heart failure, and died 28 days following the sixth cycle of R‐CHOP. The 5‐year PFS and OS rates in the nontransplant eligible group were 0.0% and 39.9%, respectively. The rates of PFS and OS were higher among the auto‐HCT eligible group compared to the non‐transplant eligible group. The median survival was 68 months in the auto‐HCT eligible group compared to 32 months in the nontransplant eligible group and the median PFS was 33 months compared to 12 months. There was no plateauing of the survival curves in either group.

## Discussion

In this study, a strategy of abbreviated R‐Hyper‐CVAD‐based induction followed by consolidative auto‐HCT was feasible in half of the newly diagnosed MCL patients. This strategy was associated with a 5‐year PFS and OS rate of 31.2% and 62.5%, respectively, without a plateauing of the survival curves (Fig. 2). This strategy provides moderate potential of long‐term survival outcomes comparable to the published literature (Table 2).

**Table 2:** Studies of first line treatments for MCL patients with auto‐HCT

Study (year of publication)	Study	Patients (*n*)	Regimen	ORR (CR)	OS (years)	PFS/DFS (years)
Khouri et al. (1998) 29	Phase II	25	HCVAD + auto‐HCT	93% (38%)	92% (3 y)	72% (3 y)
Dreyling et al. (2005) 25	Phase III	62	R‐CHOP + auto‐HCTR‐CHOP + INF	NA	83% (3 y)77% (3 y)(*P* = 0.18)	54% (3 y)25% (3 y)(*P* = 0.01)
Andersen et al. (2003) 4	Phase II	41	Maxi R‐CHOP + auto‐HCT	76% (27)	51% (4 y)	15% (4 y)
Geisler et al. (2008) 5	Phase II	160	Maxi R‐CHOP + R + Ara‐C + auto‐HCT	96% (54%)	70% (6 y)	66% (6 y)
Damon et al. (2009) 30	Phase II	78	R‐MTX‐CHOP + Ara‐C +VP16 + auto‐HCT	NA	64% (5 y)	56% (5 y)
Merli F et al. (2012) 24	Phase II	63	R‐HCVAD + auto‐HCT	83% (72%)	73% (5 y)	61% (5 y)
Van't Veer et al. (2009) 7	Phase II	66	R‐CHOPx3 + R‐Ara‐c x1 + auto‐HCT	70% (64%)	79 ± 7% (4 y)	46 ± 9% (4 y)
Delarue et al. (2013) 8	Phase II	60	CHOP X2 + R‐CHOP X1 + R‐DHAP X3 + auto‐HCT	95% (57%)CR 12% after R‐CHOP	75% (5 y)	EFS 83 months
Hermine et al. (2012) (ASH abstract) 9	Phase III	455	R‐CHOP X6 + auto‐HCTCHOPX3 + R‐DHAPX3 + auto‐HCT	97% (61%)98% (63%)	82 monthsNR(*P* = 0.045)	TTF 46 monthsTTF 88 months(*P* = 0.038)
Vandenberghe et al. (2003) 15	Retrospective	195	Auto‐HCT	(67%)	50% (5 y)	33% (5 y)
LaCasce et al. (2012) 21	Retrospective	83213429	RHCVADR‐HCVAD + auto‐HCTR‐CHOP + auto‐HCTR‐CHOP	NA	85% (3 y)87% (3 y)87% (3 y)69% (3 y)(*P* = NS)	58% (3 y)55% (3 y)56% (3 y)18% (3 y)R‐CHOP inferior to others(*P* < 0.004)
CIBMTR Data 2000–2009 (2011) 31	Retrospective	2574	Auto‐HCT	NA	71 ± 2% (3 y)	NA
Tam et al. (2009) 32	Retrospective	29137	R‐HCVAD + auto‐HCTHCVAD + auto‐HCTR‐CHOP + auto‐HCT	96% (96%)	61% (6 y)	39% (6 y)
Ottawa Hospital experience in this cohort	Retrospective	44	R‐HCVAD x2 + auto‐HCT	89% (61%)	OS 62.5% (5 y)	PFS 31.2% (5 y)

MCL, Mantle cell lymphoma; auto‐HCT, autologous hematopoietic cell transplant; ORR, overall response rate; CR, complete response; PFS, progression‐free survival; HCVAD, Hyper‐CVAD (Hyper‐CVAD + MA); R‐CHOP, rituximab, cyclophosphamide, adriamycin, vincristine, and prednisone; INF, maintenance therapy with interferon alfa; N/A, not available; maxi R‐CHOP, rituximab + augmented CHOP (maxi‐CHOP); Ara‐C, cytarabine; MTX, methotrexate; VP16, etoposide; DHAP, high dose cytarabine, cisplatin and dexamethasone; EFS, event free survival; NR, not reached; TTF, time to treatment failure.

MCL response rate, response duration, and in some instances survival have improved as a result of currently utilized chemo‐immunotherapy strategies that are variably followed by consolidative high dose therapy, maintenance therapy or observation. Despite these improvements, MCL relapse remains the norm and factors that may determine therapeutic approach include patient's age, comorbid status, disease risk stratification based on MIPI score, and the individual center's preference 14. The depth of response to induction may affect the long‐term outcomes that may be enhanced by an auto‐HCT approach 4, 15, 16. Pott and colleagues from the European MCL intergroup study found molecular remission (MR) to be an independent prognostic factor for response duration in 259 MCL patients treated in two randomized trials of the European MCL Network (MCL Younger and MCL Elderly trials) and auto‐HCT increased the proportion of patients in MR from 55% pre‐high‐dose therapy to 72% after 16.

Various chemotherapy regimens have been used as first‐line treatment for MCL, without consolidation with auto‐HCT (Table 3). The inclusion of rituximab with induction chemotherapy and/or as maintenance therapy improved the outcomes in patients treated with chemo‐immunotherapy strategies without auto‐HCT 17, 18, 19, 20). Lenz and colleagues from the German Low Grade Lymphoma Study Group (GLSG) reported the superiority of R‐CHOP compared to CHOP in a prospective randomized study in terms of ORR, CR and time to treatment failure (TTF) with no differences in PFS and OS 17. Recently, Kluin‐Nelemans and colleagues showed the superiority of R‐CHOP eight cycles compared to six cycles of rituximab, fludarabine, and cyclophosphamide (R‐FC) in patients 60 years of age or older with superior PFS and OS and less treatment‐related toxicities 18. Among responders to R‐CHOP, rituximab maintenance therapy significantly improved 4‐year OS (87%) compared to alfa interferon (INF) maintenance therapy (63%; *P *=* *0.005). In our study, we did not see a benefit with the addition of rituximab to the induction chemotherapy pretransplantation among the auto‐HCT eligible group (Fig. 3). However, this may well be related to our sample size.

**Table 3:** Studies of first line treatments for MCL patients without consolidation with auto‐HCT

Study (year of publication)	Study	Patients (*n*)	Regimen	ORR (CR)	OS (years)	PFS/ DFS (years)
Lenz et al. (2005) 17	Phase III	122	R‐CHOPCHOP	94 (34)75 (7)	*P* = 0.93	*P* = 0.31
Romaguera et al. (2005) 22	Phase II	97	HCVAD (x3‐4 cycles)	97% (87%)	82% (3 y)	64% (3 y)
Herold et al. (2008) (abstract)‐OSHO#39 trial 19	Phase III	4644	MCP + IFNR‐MCP + IFN	63% (15%)71% (32%)	56 months50 months	18 months20 months
Kluin‐Nelemans et al. (2012) 18	Phase III	560(age >60 years)	R‐CHOPX 8R‐FC X 6	86% (34%)78% (40%)(*P* = 0.06)	62%; (4 y)47% (4 y)(*P* = 0.005)	TTF 28 monthsTTF 27 months
Rummel et al. (2013) 33	Phase III	98	R‐CHOPBR	94% (30%)94% (40%)	NS	22 months33 months
Flinn et al. (2014) 34	Phase III	3334	R‐CHOP or R‐CVPBR	85% (27%)94% (50%)	NA	NA
Robak et al. (2015) 27	Phase III	244243	R‐CHOPVcR‐COP	89% (42%)92% (53%)	54% (4 y)64% (4 y)(*P* = 0.17)	14.4 months24.7 months(*P* < 0.001)

MCL, Mantle cell lymphoma; auto‐HCT, autologous hematopoietic cell transplant; ORR, overall response rate; CR, complete response; PFS, progression‐free survival; R‐CHOP, rituximab, cyclophosphamide, adriamycin, vincristine, and prednisone; HCVAD, Hyper CVAD (each cycle being one of Hyper CVAD + one of MA); R‐MCP+, rituximab + mitoxantrone, chlorambucil, and prednisolone followed by maintenance therapy with interferon alfa; R‐FC, rituximab, fludarabine, and cyclophosphamide; TTF, time to treatment failure; BR, Bendamastin and rituximab; NS, not significant; NA, not available; R‐CVP, rituximab, cyclophosphamide, vincristine, and prednisone; VcR‐COP, bortezomib (Vc), rituximab,cyclophosphamide, adriamycin, and prednisone.

The effect of the remission induction regimen on transplant outcomes is being defined. The inclusion of high dose cytarabine may improve the outcomes of ORR, Disease Free Survival (DFS) and even OS 4, 5, 6, 7, 8, 9. The Nordic Lymphoma Group showed benefit of the inclusion of high dose cytarabine with augmented CHOP (maxi‐CHOP) and rituximab prior to auto‐HCT 4, 5, 6. The 6‐year OS and PFS were 70% and 56%, respectively, with non‐relapse mortality (NRM) of 5% 5. Ultimately despite initial optimism, disease relapses occurred beyond 5 years after therapy. The MIPI and Ki‐67‐expression were the only independent prognostic factors for survival 6. Recently, Hermine and colleagues presented the final analysis of the MCL Younger Trial of the European Mantle Cell Lymphoma Network. This trial compared six courses of R‐CHOP followed by CY/TBI auto‐HCT (arm A) to alternating courses of 3x CHOP and 3x DHAP plus Rituximab followed by a high‐dose cytarabine containing conditioning regimen with TBI, cytarabine, and melphalan auto‐HCT (arm B) in 455 previously untreated patients up to age 65 9. TTF, remission duration, and OS were superior if high dose cytarabine was included in the induction regimen. Of note, however, is that the pretransplant conditioning regimens were different between the two study arms, possibly affecting the outcome measures. In contrast, recently La Casce and colleagues reported an analysis from the National Comprehensive Cancer Network (NCCN) NHL Database 21. They included patients under age 65 diagnosed between 2000 and 2008 treated with R‐Hyper CVAD alone (79% received ≥3 cycles; R‐Hyper‐CVAD + R‐MA), R‐Hyper CVAD followed by auto‐HCT (66% received <3 cycles of induction), R‐CHOP followed by auto‐HCT, or R‐CHOP alone. Cytarabine‐containing conditioning regimens were used in 18% of the R‐CHOP/auto‐HCT group and in 29% of the R‐Hyper‐CVAD/auto‐HCT group. The use of R‐Hyper‐CVAD before transplantation did not yield improved PFS compared with R‐CHOP/auto‐HCT or R‐Hyper‐CVAD alone, though the number of patients in each of the transplant arms was small. There was no difference in PFS between intensive chemotherapy regimens (*P* = 0.57), which all demonstrated superior PFS compared with R‐CHOP alone (*P* = 0.004). There was no difference in OS between the R‐Hyper‐CVAD and R‐CHOP/auto‐HCT (*P* = 0.98). R‐CHOP alone was inferior to all other strategies. In this retrospective publication, despite intensive regimens, the median PFS was 3 to 4 years.

Published data from the MD Anderson Cancer Center by Romaguera and colleagues using R‐Hyper CVAD for a total of three to four cycles (each cycle being one of R‐Hyper CVAD + one of R‐MA) in patients younger than 65 years resulted in 3‐year failure‐free survival and OS rates of 73% and 86%, respectively 22. When this regimen was studied by the Southwest Oncology Group in a multicentre setting, the 2‐year PFS and OS rates were 64% and 76% 23. The Italian Lymphoma Group study reported their multicenter study of R‐Hyper‐CVAD followed by auto‐HCT with 5‐year PFS and OS rates of 61% and 73%, respectively 24. The goal was administration of 4 cycles of treatment (R‐Hyper‐CVAD + R‐MA), though only 37% of patients completed all cycles and there was a TRM of 5%.

R‐Hyper‐CVAD alone is associated with significant hematological toxicity, with TRM of 2–5% 17, 18. We experienced two treatment‐related deaths (4.3%) during R‐Hyper‐CVAD induction. Our center adapted this induction regimen for auto‐HCT eligible patients by decreasing the number of R‐Hyper‐CVAD cycles to two (R‐ Hyper‐CVAD + R‐MA) in hopes of minimizing treatment‐related toxicity, while incorporating consolidative transplantation. Our goal was to successfully mobilize and transplant all eligible patients while maintaining chemo‐sensitivity and an adequate performance status. Of note, abbreviating the number of cycles of R‐Hyper‐CVAD allowed treatment to be completed within a timeframe comparable to 6 cycles of RCHOP. We were able to transplant 44 of 46 transplant eligible patients with this approach. Our survival outcomes are comparable to the published literature that tends to use more cycles of intensive induction therapy.

The role of consolidative auto‐HCT in MCL remains controversial as front line therapy. An increasing body of literature is accumulating that demonstrates improved response rate, PFS, and OS with this approach (Table 2). Despite adapting upfront intensive chemo‐immunotherapy with consolidative auto‐HCT, the 5‐year DFS and OS in published trials is poor, ranging from 33% to 66% and 50–75%, respectively, without an apparent plateau (Table 2). Dreyling and colleagues on behalf of the European MCL Network reported an advantage to upfront consolidative auto‐HCT in patients who responded to induction with a CHOP‐like regimen, then Dexa‐BEAM and HSC mobilization followed by CY/TBI and auto‐HCT 25. In this randomized controlled trial, patients were randomized to auto‐HCT or IFN maintenance without auto‐HCT. Auto‐HCT patients had a significantly longer PFS with a median of 39 months compared to 17 months in the IFN arm, without improvement in survival. The PFS of 39 months in this study is comparable to our PFS of 33 months in our auto‐HCT group; the PFS of the IFN group is closer to the PFS of 12 months in our nontransplanted, less aggressively treated cohort.

Outcomes in our nontransplant eligible group are poor when compared to the auto‐HCT eligible group despite the addition of rituximab maintenance therapy. The inferiority of the outcomes among non‐transplant eligible group likely affected, independently of treatment, by heterogeneity of therapy and increase in comorbidities and age. However, Nontransplant eligible patients were precluded from the intensive chemotherapy approach due to older age and/or comorbidities. Formalized geriatric and comorbidity assessments could be incorporated into the clinic so as not to inappropriately exclude older patients from potentially more effective therapies. Recently, Jantunen and colleagues reported the feasibility of auto‐HCT for MCL in elderly patients from the EBMT registry. They compared 79 patients ≥65 years of age (median 67; range 65–73) with 655 patients <65 years of age with a median age of 56 years 26. There were no significant differences at 5 years in relapse rate, PFS, NRM, and OS between the groups, demonstrating that selected elderly patients can be treated with an auto‐HCT approach. There are limitations to our study. This was a retrospective, single center analysis, with a limited number of patients in each group, lack of central pathology confirmation and lack of the impact of the proliferation index (Ki67), blastoid/pleomorphic morphology, and molecular remission assessment on the outcomes. Potentially due to our small sample size, we could not demonstrate the prognostic utility of the M‐IPI score at diagnosis among the auto‐HCT eligible group (Fig. 4).

The incorporation of novel agents as induction or maintenance therapy for newly diagnosed MCL patients was showing promising results 20, 27, 28. Recently, Robak and colleagues (LYM‐3002) reported significant improvement of PFS, with relative improvement of 59%, by substituting bortezomib (Vc) for vincristine in frontline therapy with R‐CHOP in newly diagnosed MCL patients (*n* = 487) who were ineligible or not considered for Auto‐HCT 27. This phase 3 trial showed median PFS, after median follow up of 40 months, was 24.7 months in VcR‐COP group versus 14.4 months in R‐CHOP group (HR 0.63; *P* < 0.001) with a median treatment‐free interval (40.6 months vs. 20.5 months) and the 4‐year OS rate was 64% versus 54% (HR 0.80; *P* = 0.17). Further, Chang and colleagues in ECOG (E1405) phase 2 trial reported similar 3 years PFS (72%) and OS (88%) by comparing Auto‐HCT (*n* = 22) versus MR (*n* = 44) in 75 previously untreated MCL patients who received VcR‐CVAD induction chemotherapy (rituximab, bortezomib, modified hyper‐cyclophosphamide, doxorubicin, vincristine, dexamethasone) every 21 days for six cycles that followed by consolidation therapy with MR for 2 years or Auto‐HCT in transplant‐eligible patients, with a median age of 62; range 40–76 20. In this study, the ORR for VcR‐CVAD induction chemotherapy was 95% and a CR was achieved in 68% of patients. Ruan and colleagues reported the feasibility of upfront chemotherapy‐free approach in newly diagnosed MCL patients (*n* = 38), using lenalidomide and rituximab as a combination biologic doublet for both an induction phase and a maintenance phase 28. They showed ORR of 84.2% in all patients with CR of 52.6% at a median follow‐up of 24 months.

## Conclusion

The treatment of MCL remains a challenge, but gains have been made in the last decade. Our abbreviated R‐Hyper‐CVAD based induction strategy followed by consolidative auto‐HCT is a safe and feasible treatment modality for transplant eligible patients, with outcomes comparable to the published literature. The induction regimen incorporates both Rituximab and cytarabine. The inclusion of both of these agents has been shown to improve outcomes. The poor outcome of our nontransplant eligible group highlights the need for improved therapies for this subgroup. A risk adaptive strategy incorporating formalized comorbidity assessment of the older patient is desirable, as excluding patients from high dose therapies by age alone is not acceptable. The main limitation to the current treatment approach is relapse. The development of more effective, better tolerated chemotherapy regimens and the incorporation of novel agents as induction or maintenance therapy will hopefully improve outcomes in both the transplant and the nontransplant settings.

## Conflict of Interest

None declared.
